# A facile template-free strategy for synthesizing hydroxymethyl-poly(3,4-ethylenedioxythiophene) nanospheres

**DOI:** 10.1038/s41598-020-61072-5

**Published:** 2020-03-04

**Authors:** Chanhyuk Jee, Kyung Seok Kang, Ji-Hong Bae, Hyo Jin Jung, WonBin Lim, Byeong Joo Kim, PilHo Huh

**Affiliations:** Pusan National University, Department of Polymer Science and Engineering, Busan, 609-735 South Korea

**Keywords:** Chemistry, Engineering, Materials science, Nanoscience and technology

## Abstract

Hydroxymethyl-poly(3,4-ethylenedioxythiophene)(PEDOT-OH) nanospheres self-assembled using physical blowing method, which continually used a syringe, were successfully formed through aqueous solution polymerization under the oxidative initiators. The effect of blowing on the morphological properties of PEDOT-OH was precisely evaluated based on the different amount of initiator. The concentration of ammonium persulfate might be a driving force in the self-assembly process to create the PEDOT-OH nanospheres. The electrical and electrochemical properties of the resulting nanospheres were also characterized using four-point probe and cyclic voltammetry.

## Introduction

The formation of PEDOT nanostructures with controlled morphology and size has been considered as an important branch of material research because dimensionality could be a useful factor to determine its extraordinary properties and provide a good platform in potential applications. One-dimensional (1D) nanotubes, nanofibers or nanowires of conducting polymer have been extensively prepared using either chemical-oxidative or electrochemical methods because of their promising applications in electrical and optoelectronic nanodevices^1–5^. Three-dimensional (3D) spheres of conducting polymer have also been attracted attention owing to their unique nanostructures, which may provide the specific optical, electronic, and mechanical properties. To further create different micro- and nanostructures, some studies on controlled polymerization of conducting polymers have been furtherly performed. The controlled morphologies of conducting polymers have been developed by soft templates such as DNA scaffolds^6^, amphiphilic peptides^7^, small molecules^8^, biomembrances^9^, and cellulose derivatives^10^ as well as hard templates such as anodic aluminum oxide^11^, mesoporous silica^12^, hydrogel bio electronics^13–15^ and actuator^16^. On the other hand, there are a few reports on one-step synthetic strategies to produce PEDOT 3D nanostructures.

In this study, a facile one-pot synthesis of PEDOT-OH nanospheres without any surfactants and templates could be successfully formed by just controlling with/without blowing condition and the quantity of initiator. The as-synthesized PEDOT-OH nanospheres exhibited some interesting electrical and electrochemical properties for potential applications. Central challenge in this work is to create the well-defined PEDOT-OH nanospheres based on a one-step process. The novel PEDOT-OH nanospheres are also designed to analyze the three-dimensional shape effect for electrical properties, in comparison with one or two-dimensional shapes..

## Results

**Figure 1:**
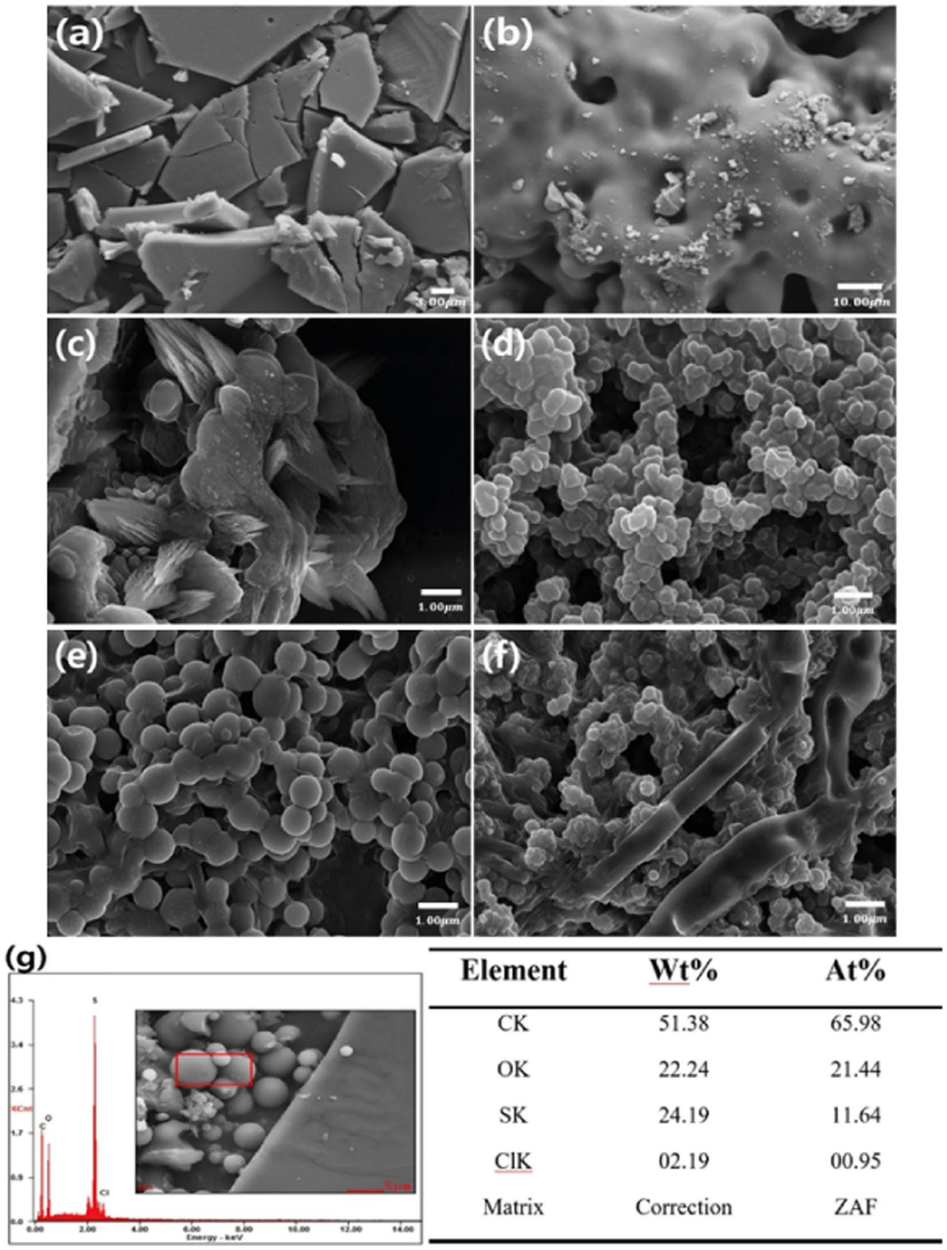
Morphology of PEDOT-OH: (**a**) without a blowing method during polymerization, (**b**) polymerization with a blowing method.(used APS initiator quantity is 0.03 g.) FE-SEM images of chemical polymerization of PEDOT-OH with different initiator (APS) quantities ((**c**) 0.001 g, (**d**) 0.005 g, (**e**) 0.03 g, (**f**) 0.05 g) under the same conditions (monomer (hydroxymethyl-EDOT) 0.05 g, HCl 1 ml, solvent (ethanol:H_2_O = 7:3 5 ml), room temperature and 12 h. synthesis times with blowing for 1 time/h. a total of 12 times during synthesis. Energy dispersive spectrometry (EDS) graph and data (**g**) of PEDOT-OH spheres.

**Figure 2:**
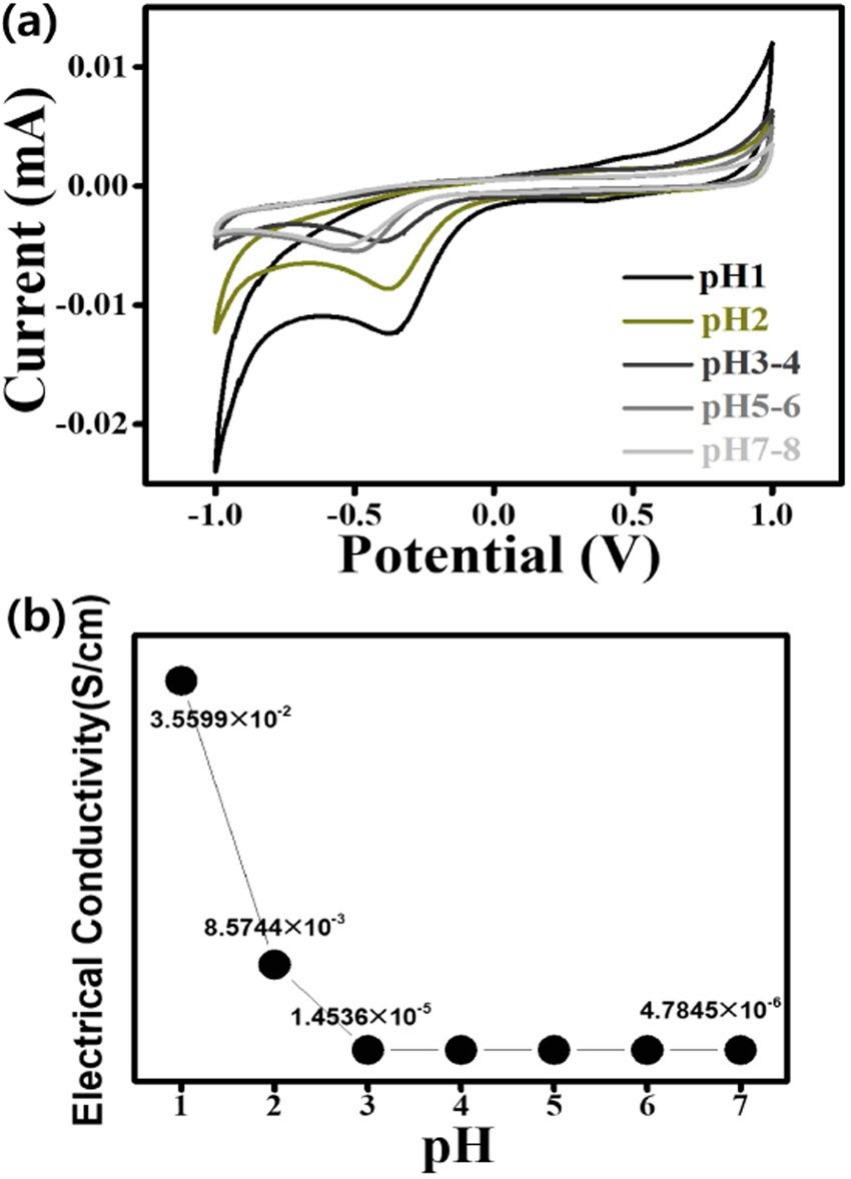
Electrical properties of hydroxymethyl-poly(3,4-ethylenedioxythiophene (PEDOT-OH) nanospheres (Fig. 1(e)), changing from base to acid states. Cyclic Voltammetry is recorded at scan rate 20 mV/s with a 0.001 cm^2^ carbon working electrode, a 0.001 cm^2^ Pd counter electrode, and a Ag/AgCl reference electrode. (**a**) Cyclic voltammetry and (**b**) electrical conductivity according to acidity.

Figure 1 displays a schematic mechanism to create the PEDOT-OH nanospheres based on a blowing process with different initiator concentrations. PEDOT-OH macrostructures using only APS without blowing were pseudo-plate in shape and quite non-uniform in size, as shown Fig. 2(a). A tremendous change in the PEDOT-OH nanostructures was observed from plate to sphere when the blowing procedure was introduced to the reaction system, as shown in Fig. 1(a,b). One expected formation mechanism of Fig. 1(c–f) is that micelles composed of blowing-bubble/PEDOT-OH/HCl might be induced in the reaction solution due to the hydrophilic interactions between the bubble and PEDOT-OH/HCl. Moreover, APS might be well dispersed and anchored on the surface of the spherical bubbles due to the hydrophilic groups. The blowing-bubble/PEDOT-OH/HCl micelles of the reaction might be regarded as templates in the formation of nanospheres through a self-assembly process. Figure 1 shows a typical SEM images of PEDOT-OH nanospheres prepared as a function of APS initiator and blowing at a fixed rate using a syringe. The as-prepared PEDOT-OH nanostructures exhibited changes morphologically from 1D flat to 3D spheres or 3D cylinders, with increasing the added initiator. The PEDOT-OH nanospheres formed in this study had the average diameters ranging from 300 nm to 500 nm when the amount of APS was changed from 0.001 to 0.05 g, as shown in Fig. 1(c,d). The results indicated that the concentration of APS might have a great influence on the morphology of the resulting PEDOT-OH nanostructures. When the amount of APS was lower than 0.001 g, the nanospheres in shape were the dominant morphology, whereas a cylindrical shape was observed predominantly when the quantity of APS was higher than 0.05 g. This indicates that hydrogen-bond interaction might be the main driving force for the formation of self-assembled PEDOT-OH nanospheres. Moreover, the nanospheres were the dominantly morphological structure in shape when the APS concentration was 0.03 g at a fixed syringe blowing rate. The PEDOT-OH nanospheres exhibited a uniform thickness distribution with an average diameter of 500 nm. In comparison with different concentration of initiator, the PEDOT-OH nanospheres synthesized using 0.03 g APS exhibited a good regular morphology. The EDAX patterns in Fig. 1(g) clearly show the presence of PEDOT-OH nanospheres composed of S, C, and O.

The electrochemical properties of the as-prepared PEDOT-OH nanospheres under 0.1 M PBS containing 1.0 mM H_2_O_2_ were analyzed by the cyclic voltammetry series of Fig. 2(a), at a pH ranging from 1 to 8. As shown in Fig. 2(a), the individual CV curve obtained at different pH showed two major current responses for PEDOT-OH nanospheres. The well-defined large peak at approximately −0.35 V might be assigned to the reduction of PEDOT-OH units in the nanosphere, while the peak occurred at about +0.5 V could be assigned to the oxidation of PEDOT-OH nanospheres. Upon titrating between acid and base forms, the positions of the redox peaks shifted slightly with pH, but their absorbance shapes were almost similar regardless of pH. The decreased area of redox absorbance depending on pH might be explained by the decrease in band gap, which is in agreement with results of the electrical conductivity measurements.

Figure 2(b) shows the conductivities of the PEDOT-OH nanospheres as a function of pH doping level. The conductivity values increased significantly with increasing acid concentration. The average conductivity of PEDOT-OH nanospheres doped at pH 1.0 was approximately 3.56 × 10^−2^ S/cm. Under base and acid conditions, the conductivities grew almost exponentially by an order of two along the pH level investigated. This phenomenon may be explained by the formation of sufficient complexes between the acids and nanospheres surfaces, because of the increased surface area compared to the other morphologies. This result well corresponds to the good electrochemical activities of PEDOT-OH nanospheres doped at pH < 7.0, which may provide good protonation and abundance of cationic states upon oxidation.

## Discussion

PEDOT-OH nanospheres were self-assembled using a complex method, including chemically oxidative polymerization and physical blowing. The hydrogen bond may act as driving force for the self-assembly of nanospheres. PEDOT-OH nanospheres may be formed on the monomer-acid interface during the injection of physical bubbling. The electrochemical and electrical properties of the PEDOT-OH nanospheres may allow the practical applications in various electro-fields requiring an electroactive substrate. And supplementary file with further informations about this experiment were provided in this article.

## Methods

**Scheme 1:**
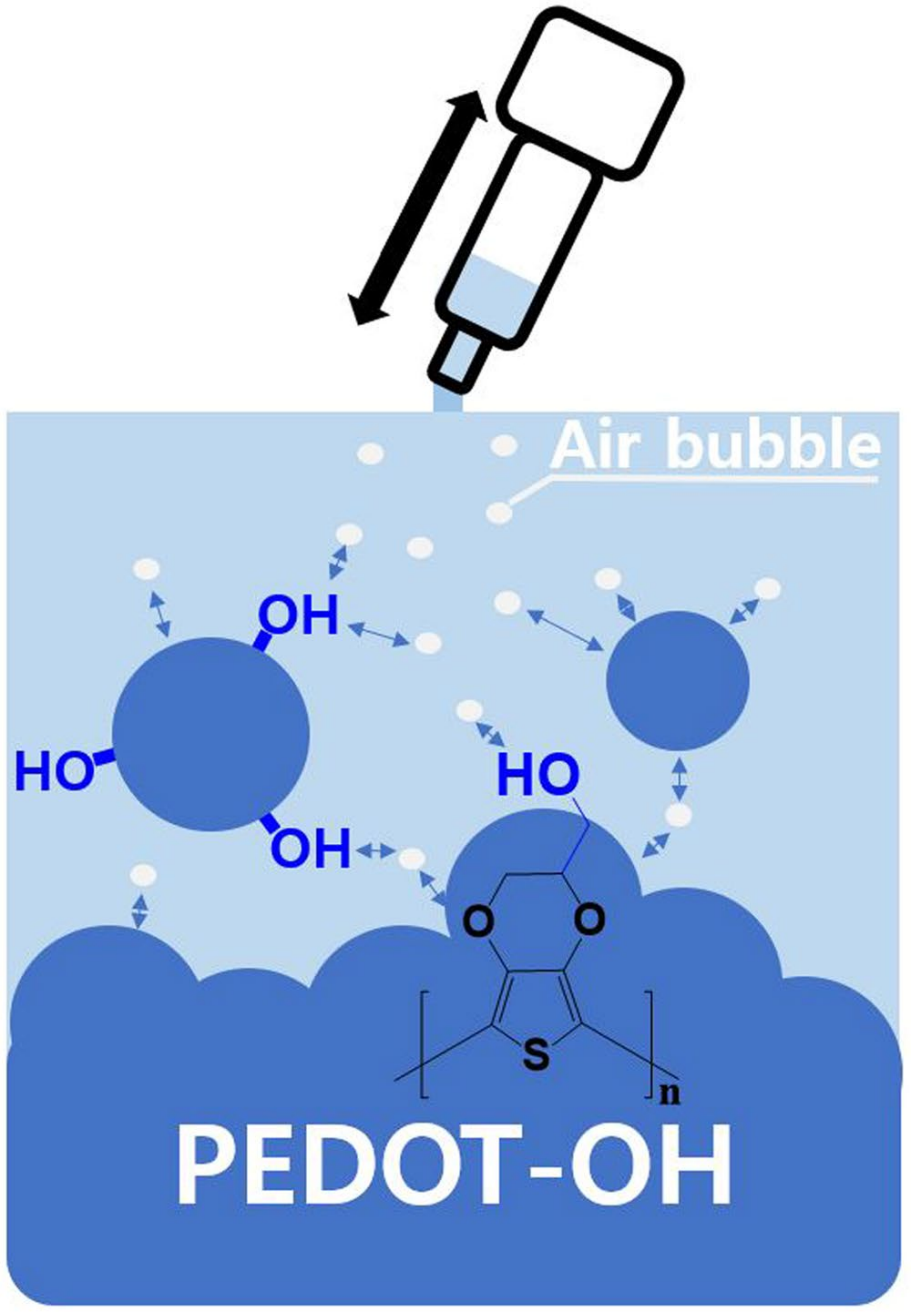
A schematically synthetic concept for creating the sphere morphology of PEDOT-OH onto the interfaces of air bubbles by hydrogen bonding based on a blowing method.

Scheme 1 presents a schematic diagram of the blowing procedure to form PEDOT-OH nanospheres on one-step oxidative chemical polymerization. 0.05 g of hydroxymethyl-EDOT was dissolved in 5 mL of an ethanol:H_2_O = [7:3] solution (pH = ~1) with stirring. Subsequently, (NH_4_)_2_S_2_O_8_ (APS) was dropped successively into the mixture with vigorously stirring for 12 h at room temperature, ranging from 0.001 g to 0.05 g. The syringe blowing step to form the nanospheres was carried out periodically 12 times over 12 h under air condition. The resulting PEDOT-OH nanostructures were filtered, rinsed, and dried on a petri dish for 24 h at 40 °C.
